# Iodine-131 and Thyroid Function: Ostroumova et al. Respond

**DOI:** 10.1289/ehp.1307737R

**Published:** 2014-02-01

**Authors:** Evgenia Ostroumova, Alexander Rozhko, Maureen Hatch, Kyoji Furukawa, Olga Polyanskaya, Robert J. McConnell, Eldar Nadyrov, Sergey Petrenko, George Romanov, Vasilina Yauseyenka, Vladimir Drozdovitch, Viktor Minenko, Alexander Prokopovich, Irina Savasteeva, Lydia B. Zablotska, Kiyohiko Mabuchi, Alina V. Brenner

**Affiliations:** 1 Division of Cancer Epidemiology and Genetics, National Cancer Institute, National Institutes of Health, Department of Health and Human Services, Bethesda, Maryland, USA; 2 The Republican Research Center for Radiation Medicine and Human Ecology, Gomel, Belarus; 3 Department of Statistics, Radiation Effects Research Foundation, Hiroshima, Japan; 4 The Thyroid Center, Columbia University, New York, New York, USA; 5 Department of Anthropoecology and Epidemiology, International Sakharov Environmental University, Minsk, Belarus; 6 Belarusian Medical Academy of Post-Graduate Education, Minsk, Belarus; 7 Department of Epidemiology and Biostatistics, University of California, San Francisco, San Francisco, California, USA

Sun’s comments about the relationship between iodine-131 (^131^I), hypothyroidism, and simple diffuse goiter suggest a misunderstanding of our study findings. We reported a significantly higher—rather than lower—radiation-associated risk of hypothyroidism among study participants without goiter than in the participants with goiter (Ostroumova et al. 2013). Specifically, the excess odds ratio (EOR) per Gray of ^131^I thyroid dose was 0.50 [95% confidence interval (CI): 0.24, 0.90] in participants without goiter and 0.04 (95% CI: –0.09, 0.32) in those with goiter. We also reported a lack of significant variation of EOR per Gray for hypothyroidism by levels of urinary iodine (*p* = 0.23), although in the discussion we noted that iodine concentration in spot urine samples, unlike presence of diffuse goiter, reflects current levels of iodine intake and is subject to high within-individual variability.

The territories of Belarus were known to be iodine deficient before the Chernobyl accident; in the Soviet Union there was a system of iodine prophylaxis that was discontinued by the mid-1980s (Kholodova and Fedorova 1992). In 1995–1998, five of the six Belarus regions were classified as having moderate iodine deficiency, whereas the Gomel region, most heavily contaminated with ^131^I, was classified as having mild iodine deficiency partly due to some iodine supplementation in this area after the Chernobyl accident (Arinchin et al. 2000). High prevalence of diffuse goiter detected by ultrasound in children and adolescents in the relatively uncontaminated Brest region (27.8%) and low prevalence in the heavily contaminated Gomel region (5.6%) (Arinchin et al. 2000) support the idea that these differences are attributed to different intake of dietary iodine and not to ^131^I exposure. Moreover, there is little evidence of a dose–response association between thyroid exposure and simple diffuse goiter in other radiation-exposed cohorts (Ron and Brenner 2010).

As we described in the “Materials and Methods” of our article (Ostroumova et al. 2013), availability of individual direct measurements of thyroid radioactivity served as a key criterion for inclusion into the study. All study participants had direct measurements of thyroid radioactivity performed within 2 months after the accident. In the methods for dosimetry, we cited the article by Drozdovitch et al. (2013), in which dose reconstruction methods were described in detail. We also noted that intake of ^131^I on average accounted for about 95% of the estimated thyroid dose, whereas the contribution of other short-lived radioiodines, external exposures, and internal exposure from cesium-137 and cesium-134 was minor (Bouville et al. 2007).

We appreciate Sun’s interest in our study and hope our response is useful.
